# FAMILY CAREGIVERS’ REFLECTIONS ON FAMILY SUPPORT AND PREPAREDNESS FOR END-OF-LIFE CAREGIVING

**DOI:** 10.1093/geroni/igad104.1373

**Published:** 2023-12-21

**Authors:** Aimee Fox, Christine Fruhauf, Julia Sharp

**Affiliations:** Utah Valley University, Utah, United States; Colorado State University, Fort Collins, Colorado, United States; Colorado State University, Fort Collins, Colorado, United States

## Abstract

Family caregivers often report not having the knowledge or skills to provide care needed at their care receiver’s end of life (EoL), leaving them vulnerable to emotional, psychological, physical, and financial stress. These stressors may be due to increasingly complex caregiving responsibilities, difficult medical decisions, and anticipatory grief. Studies have also found a higher incidence of adverse effects among dementia caregivers. Few studies have examined how family support is associated with preparedness for EoL caregiving. Thus, the purpose of this study was to explore family caregivers’ feelings of preparedness and family-member support for EoL caregiving, comparing responses between dementia care providers with all other caregivers. A sample of family caregivers were recruited to complete an online, self-report survey. Open-ended questions were included to explore caregivers’ feelings of preparedness and family support. Of all participants, 206 family caregivers responded to the open-ended questions. Nearly 40% (n=79) of these caregivers were providing care to a family member with Alzheimer’s disease or related dementia. Qualitative methods were used to analyze data, develop a coding scheme, and identify themes. Findings suggest that most family caregivers are providing care without adequate help, support, or communication from other family members. This lack of support seems to leave dementia caregivers feeling less prepared and more frustrated than other caregivers. Our findings highlight the importance of family communication about advance care planning and caregiving responsibilities to promote family caregiver preparedness and well-being, and effective care and quality of life for an individual at the end of their life.

